# Neural Patterns of Reorganization after Intensive Robot-Assisted Virtual Reality Therapy and Repetitive Task Practice in Patients with Chronic Stroke

**DOI:** 10.3389/fneur.2017.00452

**Published:** 2017-09-04

**Authors:** Soha Saleh, Gerard Fluet, Qinyin Qiu, Alma Merians, Sergei V. Adamovich, Eugene Tunik

**Affiliations:** ^1^Human Performance and Engineering Research, Kessler Foundation, West Orange, NJ, United States; ^2^Department of Rehabilitation and Movement Science, Rutgers University, Newark, NJ, United States; ^3^Department of Biomedical Engineering, NJIT, Newark, NJ, United States; ^4^Department of Physical Therapy, Movement, and Rehabilitation Science, Northeastern University, Boston, MA, United States; ^5^Department of Bioengineering, Northeastern University, Boston, MA, United States; ^6^Department of Biology, Northeastern University, Boston, MA, United States

**Keywords:** stroke, virtual reality, rehabilitation, motor control and learning/plasticity, functional magnetic resonance imaging neuroimaging, connectivity analysis

## Abstract

Several approaches to rehabilitation of the hand following a stroke have emerged over the last two decades. These treatments, including repetitive task practice (RTP), robotically assisted rehabilitation and virtual rehabilitation activities, produce improvements in hand function but have yet to reinstate function to pre-stroke levels—which likely depends on developing the therapies to impact cortical reorganization in a manner that favors or supports recovery. Understanding cortical reorganization that underlies the above interventions is therefore critical to inform how such therapies can be utilized and improved and is the focus of the current investigation. Specifically, we compare neural reorganization elicited in stroke patients participating in two interventions: a hybrid of robot-assisted virtual reality (RAVR) rehabilitation training and a program of RTP training. Ten chronic stroke subjects participated in eight 3-h sessions of RAVR therapy. Another group of nine stroke subjects participated in eight sessions of matched RTP therapy. Functional magnetic resonance imaging (fMRI) data were acquired during paretic hand movement, before and after training. We compared the difference between groups and sessions (before and after training) in terms of BOLD intensity, laterality index of activation in sensorimotor areas, and the effective connectivity between ipsilesional motor cortex (iMC), contralesional motor cortex, ipsilesional primary somatosensory cortex (iS1), ipsilesional ventral premotor area (iPMv), and ipsilesional supplementary motor area. Last, we analyzed the relationship between changes in fMRI data and functional improvement measured by the Jebsen Taylor Hand Function Test (JTHFT), in an attempt to identify how neurophysiological changes are related to motor improvement. Subjects in both groups demonstrated motor recovery after training, but fMRI data revealed RAVR-specific changes in neural reorganization patterns. First, BOLD signal in multiple regions of interest was reduced and re-lateralized to the ipsilesional side. Second, these changes correlated with improvement in JTHFT scores. Our findings suggest that RAVR training may lead to different neurophysiological changes when compared with traditional therapy. This effect may be attributed to the influence that augmented visual and haptic feedback during RAVR training exerts over higher-order somatosensory and visuomotor areas.

## Introduction

Recovery of hand function is challenging after stroke. Empirical data suggest that treatment can be beneficial if it includes many repetitions of challenging and meaningful tasks (1–3). Several approaches to delivering high volume, intense, and salient rehabilitation activities have emerged over the last two decades. These treatments, which include repetitive task practice (RTP), robotically assisted rehabilitation, and virtual rehabilitation activities, produce improvements in hand function that exceed the standard of care in the US (4, 5).

Although a strong case has been made that virtual reality (VR) and robotics can be useful technologies for delivering challenging, meaningful, and mass practice, outcome studies investigating the true benefits of VR/robotics as compared to dose-matched RTP remain mixed (6, 7). For example, we have shown significant group-level improvement in hand and arm function of chronic stroke survivors in response to RTP and robot-assisted VR (RAVR) training to be similar for both groups (8), a finding that agrees with group-level effects in other clinical studies (9, 10). However, whether the underlying neural patterns of reorganization that are induced by the different training regimes are also similar remains unknown. This becomes important to understand because it may inform researchers and clinicians whether RAVR versus RTP may preferentially facilitate distinct neural patterns of reorganization. If so, then perhaps the therapy choice can be tailored more appropriately to individuals to elicit optimal benefits.

The goal of this study was to compare the effect of RAVR- and RTP-based interventions on neural pattern reorganization. Because neural reorganization likely reflects complex processes that include the formation of new connections and/or re-weighting of existing connections, the patterns that emerge are unlikely to be reliably captured using one proxy of activation. For example, while numerous studies have shown training-induced changes in the extent of brain activity, the results of those studies conflict in terms of whether the changes reflect an increase or a decrease in brain activity (11–15). Second, there seems to be a relationship between the pattern of reorganization (increase or decrease in ipsilesional somatosensory activation) and intactness of the hand knob area of M1 and its descending motor fibers (16), and a dependence on whether the lesion is cortical or subcortical (17). Connectivity measures may be a complementary way to understand neural reorganization patterns underlying stroke recovery (18) by providing additional information about dynamic network-level changes above and beyond what can be inferred from extent and laterality of activation (19, 20).

In this study, we therefore characterize the pattern of neural reorganization using multiple measures that included the magnitude of change in brain activation, the extent of activation, the re-lateralization of brain activation in a set of homologous interhemispheric regions of interest, and interactions between multiple regions of interest based on measures of functional and effective connectivity. To our knowledge, this is the first study to characterize brain reorganization at the ROI and network interaction level with multiple functional magnetic resonance imaging (fMRI) measures before and after RAVR and RTP training. In order to delineate the relevance of brain reorganization after training, we also correlated the brain activation outcomes with clinical outcome measures.

We hypothesized that both treatments might have similar effects on the magnitude and laterality of activation in a given region of interest. However, because RAVR training provides a training environment that is enriched and augmented with visual and haptic feedback, we expected that the functional and effective connectivity between motor/premotor cortices and visuomotor areas like the superior parietal lobule may show stronger effects in the RAVR group, as compared to the RTP-based training group (21–25). We propose that identifying the neurophysiologic correlates of behavioral motor function improvement might allow strategic refinement of existing training approaches and the development of individually tailored interventions.

## Materials and Methods

### Subjects

Data for a total of 19 subjects, who participated in a previous non-randomized controlled trial (8) and who were eligible for MRI, were analyzed to study neural reorganization associated with each type of therapy. Ten subjects participated in the RAVR group (2F, mean age ±1 SD: 59.6 ± 10.6 years), and nine subjects participated in the RTP group (3F, 57 ± 12.8 years). Table 1 shows subjects’ demographics and baseline clinical information, including a sum of the Modified Ashworth Scale scores for elbow flexors, wrist flexors, and finger flexors (26), and Chedoke-McMaster Impairment Inventory stages for the arm and hand (27, 28). All subjects had ischemic stroke except S7 in the RTP group, whose stroke was hemorrhagic. Inclusion criteria included (1) age between 18 and 80 years, (2) chronic phase of stroke (>6 months), (3) at least 20° of active wrist extension, (4) at least 10° of active finger extension, and (5) meeting safety standards for participating in fMRI. Exclusion criteria included (1) aphasia that would limit the ability to follow verbal instructions, (2) spatial neglect rendering a patient unable to interact with a 28-inch wide workspace and with the robot safely, and (3) participation in other upper extremity therapies during the study. Subjects for the RTP group of the study were recruited consecutively in a period of few months, while subjects in the RAVR group were chosen from a larger sample of subjects participating in a study of virtually simulated UE rehabilitation based on eligibility to participate in MRI testing.

**Table 1:** Subjects’ clinical information.

Group	Subject	Months since CVA	CVA side	CMA	CMH	Ashworth	Lesion location	Lesion volume (mm^3^)
Virtual reality (VR)	1	53	R	6	4	2	C; frontal and parietal lobes	546
VR	2	41	L	5	4	7	S; thalamic nuclei	49,280
VR	3	11	R	6	2	1	C; frontal lobe	3,960
VR	4	96	L	7	5	1	S; corona radiata	145
VR	5	132	R	5	4	3	C; frontal, parietal and temporal lobes	1,739
VR	6	96	L	4	3	1	S; pons	672
VR	7	90	L	6	0	1	C; occipital lobe	1,120
VR	8	18	R	5	4	6	S; pons	34,728
VR	9	144	L	6	6	2	S; pons	495
VR	10	15	L	4	5	5	S; thalamic nuclei	49,005

Mean (SD)		70 (±49)		5.4 (±1)	3.7 (±1.7)	2.9 (±2.3)		14,169 (±21,211)

Repetitive task practice (RTP)	1	157	L	6	6	2	S; pons	546
RTP	2	73	L	6	6	0	C; frontal, parietal and temporal lobes	4,420
RTP	3	9	R	4	4	8	S; pons	145
RTP	4	57	R	5	5	5	S; thalamic nuclei	672
RTP	5	96	L	7	5	1	S: corona radiata	1,120
RTP	6	144	L	4	4	7	C; frontal, parietal and temporal lobe	34,728
RTP	7	120	R	6	6	0	C; parietal lobe	76,966
RTP	8	145	R	4	4	1	C; frontal, parietal and temporal lobe	42,455
RTP	9	36	L	4	4	1	S; pons	37
Mean (SD)		90(± 53)		5.1 (±1.2)	4.9 (±0.9)	2.7 (±3.1)		17,898 (±27,546)

*CVA stands for cerebro-vascular accident. CMA stands for Chedoke-McMaster scale of arm movement, and CMH is the score for hand movement. L, left; R, right; S, subcortical; C, cortical*.

### Interventions

Subjects in both groups were trained 4 days a week, 3 h per day, for 2 weeks. Every effort was made to match to the dosage of training in both groups and to adapt the level of difficulty to each individual’s performance within and across sessions so that all subjects remained engaged and appropriately challenged.

#### RAVR Group

**Figure 1:**
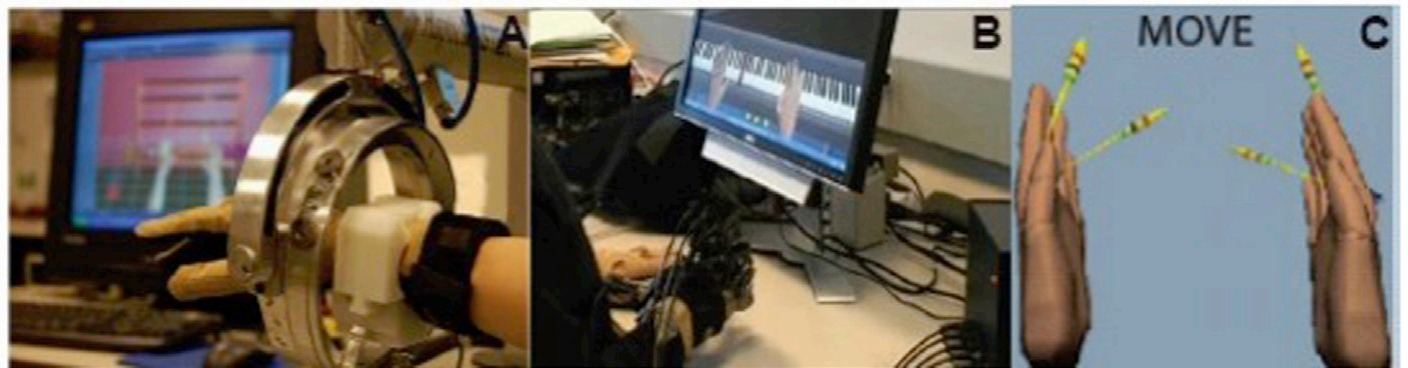
**(A,B)** The robotic arm, a data glove and force-reflecting hand system used in the robot-assisted virtual reality therapy. **(C)** Virtual reality feedback during the fMRI movement task. For each hand, one arrow points to the starting position of the hand (open) and another arrow defines the magnitude of finger flexion during the task.

Subjects participated in a 2-week training program known as New Jersey Institute of Technology RAVR (NJIT-RAVR) training. Training involved reaching for and interacting with stationary and moving virtual targets and objects in 3D space (Figure 1). Robot assistance was tailored to the individual needs of each patient and was dynamically monitored by sensor instrumentation on the robot during each movement trial. An algorithm was used to adjust the assistance based on real-time evaluation of hand velocity or active force, i.e., if the subject did not produce movement (based on recorded velocity and force) above predefined thresholds, or did not respond within 5 s of movement cue onset, the robot was actuated to provide assistance. The NJIT-RAVR intervention is explained in greater details in our previous publications (29, 30).

#### RTP Group

The 2-week training was designed based on the shaping component from the EXCITE trial (31) and a taxonomy of activities (32). Training included “reach and grasp” exercises and functional tasks. Some of the tasks required unilateral movements (e.g., coin sorting, writing, carrying objects, feeding, and using a calculator), and others required bilateral hand and arm coordination (e.g., cooking, dressing, and building with small and large objects). The RTP training is described in more details in Ref. (8).

#### Outcome Measures

Outcome measures included a battery of clinical tests to characterize functional changes and fMRI to characterize neural patterns of reorganization. The main outcome measurements were collected 1 day before the start of training (pre-test) and 1 day after the end of training (post-test).

#### Clinical Tests

Jebsen Taylor Hand Function Test (JTHFT) (33, 34) was used in quantifying motor function. The test is widely used in the literature and has excellent validity and reliability (35, 36).

#### Neurophysiology Test

Functional magnetic resonance imaging data were collected before and after subjects’ enrollment in the interventions, using the same protocols in both groups; data acquisition and outcome measures are described later.

### MRI Data Acquisition

MRI data were acquired using a 3-T Siemens Allegra head-only scanner with a Siemens standard head coil. High-resolution structural images (T1-weighted images) were acquired using Magnetization-Prepared Rapid Gradient-Echo sequence (37), with TR = 2 s, TE = 4.38 ms, voxel size 0.938 × 0.938 × 1, 176 slices, and 1 mm slice thickness. T2-weighted images (echo planar imaging sequence) were acquired with TR = 2s, TE = 30 ms, FOV 220 mm × 220 mm, voxel size = 3.4 mm × 3.4 mm × 4 mm, 32 slices, and 62 ms inter-slice time.

### Task during fMRI

During the fMRI experiments, subjects performed whole hand finger flexion with the paretic hand. Initiation and termination cues were provided by displaying the text cues “Go” or “Rest,” respectively. Two MRI-compatible fiber-optic instrumented gloves (5DT) were used to record 14 joint angles of both hands in real time to track finger flexion. Streaming data from the data gloves animated VR hand models displayed on the screen, thereby providing subjects with real-time visual feedback of their movement (38, 39). Subjects were cued to flex their fingers to meet a set of two visual targets, angled at 40 and 80% of their maximum range of motion, in a VR simulation. Prior to the experiment, each subject’s active range of finger motion was evaluated in order to set the targets’ angle properly. On each virtual hand, one arrow pointed to the starting position (hand open) and another pointed to the target (either 40 or 80% of the active range of motion). The starting position target prompted the subjects to return to the same starting position after each trial, and the goal target ensured that subjects were engaged in the experiment and performed consistent movements (Figure 1C). Non-paretic hand movement was also recorded to identify any uninstructed or mirror movements that could confound the fMRI results. The task trials’ (16 trials per target, 32 total) duration was 3 s; the trials were randomly interleaved within each functional run with inter-trial rest periods jittered and ranging in duration from 3 to 7 s. Data were collected from three functional runs for all subjects except two subjects who got tired after the second run. A total of 156 volumes were collected in each functional run.

### fMRI Data Analysis

#### Preprocessing

Functional magnetic resonance imaging data were preprocessed and analyzed using the Matlab^®^-based statistical parameter modeling software (SPM8; revision 4667). Structural and functional volumes of subjects with a left side lesion were flipped before preprocessing so that all subjects had a right-sided virtual lesion, enabling group-level averaging and statistics. Each subject’s functional volume was manually re-oriented to the AC-PC line, realigned to the first volume, and co-registered with the structural image. Realignment to the first volume showed that motion did not exceed 3 mm in all *X, Y*, or *Z* directions, in all subjects’ datasets; therefore, no data were excluded based on motion artifacts. Stroke lesions were mapped in MRIcron using the high-resolution structural image, and unified segmentation (40) was used to segment structural images after masking out the lesioned area. Normalization of the data was optimized by using the DARTEL toolbox (41) consistent with established approaches and using cost function masking (40–43). Functional images were smoothed using an 8-mm full width at half maximum Gaussian kernel.

#### General Linear Modeling (GLM) Analysis

Data of the two testing sessions, before and after therapy, were incorporated in one GLM to correlate brain activity with the hand movement functional task. Movement kinematics (movement amplitude and movement duration) were analyzed, and trials in which subjects did not comply with task instructions were excluded. Trial onset and duration were defined based on recorded finger movement data instead of onset and duration of movement cues.

#### Magnitude of Activation

Regression maps based on GLM analysis were analyzed, and the main contrasts of interest included (a) move > rest in the pre-test session, (b) move > rest in the post-test session, (c) post-test > pre-test, (d) pre-test > post-test, and (e) effect of interest (*F* test). The contrasts were done within a region of interest that included gray matter.

#### Interhemispheric Balance

Laterality index (LI) is a measure of balance in brain activity between the two brain hemispheres; a value of 1 indicates the complete dominance of the contralesional (left) hemisphere and a value of −1 indicates complete dominance of the ipsilesional (right) hemisphere (44, 45). Interhemispheric balance was computed using the LI toolbox and included the sensorimotor areas (pre-central + post-central gyri) as a region of interest (46). The following equation shows the formula used to calculate LI:

$$\text{LI}=\frac{\text{contralesional hemisphere \# of voxels}-\text{ipsilesional hemisphere \# of voxels}}{\text{contralesional hemisphere \# of voxels}+\text{ipsilesional hemisphere \# of voxels}}$$

#### Functional Connectivity of Ipsilesional Motor Cortex (iMC)

Changes in functional connectivity between the iMC and the rest of the brain were investigated using generalized psychophysiological interaction (gPPI) (47) analysis. The seed voxel for gPPI analysis was defined as the most active cluster (eight voxels within the cluster) in the iMC (contralateral to the moving hand). This analysis was used to compare the regression maps of iMC before and after therapy (in both groups): (a) regression map post-test > regression map pre-test and (b) regression map pretest > regression map post-test.

#### Effective Connectivity

Functional connectivity measures the correlation among brain regions regardless of the direction of influence, while effective connectivity measures the dynamic (time dependent) influence of one region on the other (48). Effective connectivity between bilateral motor areas and unidirectional connectivity from ipsilesional premotor, supplementary motor, and somatosensory areas to ipsilesional MC was analyzed using dynamic causal modeling (49, 50). The regions of interests were picked from the average move > rest contrast in each group. One model was created, assuming bilateral connectivity between contralesional motor cortex (cMC) and iMC, and unilateral modulatory input from ipsilesional premotor (iPMv), ipsilesional primary sensory (iS1), and ipsilesional supplementary motor area (iSMA) to iMC. This full model was estimated (in SPM8, DCM10) for each of the datasets. DCM A parameters represent the endogenous connectivity between nodes in the full model, and B parameters represent the modulation (increase or decrease) of this endogenous connectivity during the moving task. A and B parameters were added, and the DCM (A + B) parameters were compared between the two testing days and between the two groups using non-parametric Mann–Whitney *U* test. Subject 8 in the RTP group was excluded from this analysis because his brain lesion involved major ipsilesional hemisphere ROIs included in the DCM analysis.

#### Statistical Tests

Non-parametric statistical tests were used to compare the changes in outcome measures. The statistical non-parametric modeling toolbox (51, 52) was used to compare the change in brain activity after training in each group. Possible interaction between training group and training effect was tested using the Sandwich Estimator (SwE) toolbox (53), which is a tool for longitudinal and repeated measures fMRI data. Statistical threshold was set at *p* < 0.05 with family-wise error correction. Change in LI, the extent of brain activity, and DCM parameters were studied using the non-parametric Mann–Whitney *U* test in STATVIEW software. Within group, between sessions paired comparison tests were performed using Wilcoxon signed rank test. The statistical threshold in the Mann–Whitney *U* test and Wilcoxon signed rank test was set at *p* < 0.05. The relationship between changes in neurophysiology (extent of activity, lateralization, and connectivity) and (1) lesion volume, (2) performance after training based on JTHFT, and (3) ratio of change in JTHFT after training (post-test score–pre-test score) normalized to the pre-test score was studied using a non-parametric Spearman rank correlation test. Regression analysis was performed using the STATVIEW toolbox as well, and statistical threshold was set at *p* < 0.05.

## Results

### Clinical

The main outcomes of the JTHF clinical test are shown in Table 2. At baseline, there was no statistical difference in the JTHFT between the two groups (*Z* = −0.41, *p* = 0.68). Both groups showed improvement in the JTHFT score, with the percentage change reaching statistical significance in the RAVR (*Z* = −2.8, *p* = 0.005) but not in the RTP group. There was no significant between-group difference in the change in JTHFT scores.

**Table 2:** Percent change in Jebsen Taylor Hand Function Test (JTHFT) score in both groups.

Group	Subject	JTHFT% diff
Virtual reality (VR)	1	03.9
VR	2	09.7
VR	3	11.1
VR	4	30.7
VR	5	06.4
VR	6	13.9
VR	7	10.3
VR	8	10.0
VR	9	16.2
VR	10	03.2

Mean (SD)		11.54 (±7.86)

Repetitive task practice (RTP)	1	09.5
RTP	2	−03.2
RTP	3	27.5
RTP	4	19.6
RTP	5	17.5
RTP	6	23.9
RTP	7	−06.1
RTP	8	29.4
RTP	9	−23.6
Mean (SD)		10.5 (±17.97)

### Movement Performance during fMRI

Repeated measures ANOVA showed no significant difference in movement kinematics (movement amplitude and duration) during the fMRI task between the two testing days and between the groups, suggesting that subjects performed similarly consistent movements during fMRI. Thus, any differences in brain activation (across days or groups) are unlikely to be attributed to in-scan performance but rather due to training-induced patterns in activation.

### Between-Group and Within-Group Comparisons of fMRI Data across Sessions

#### Change in Extent and Magnitude of Activation

**Figure 2:**
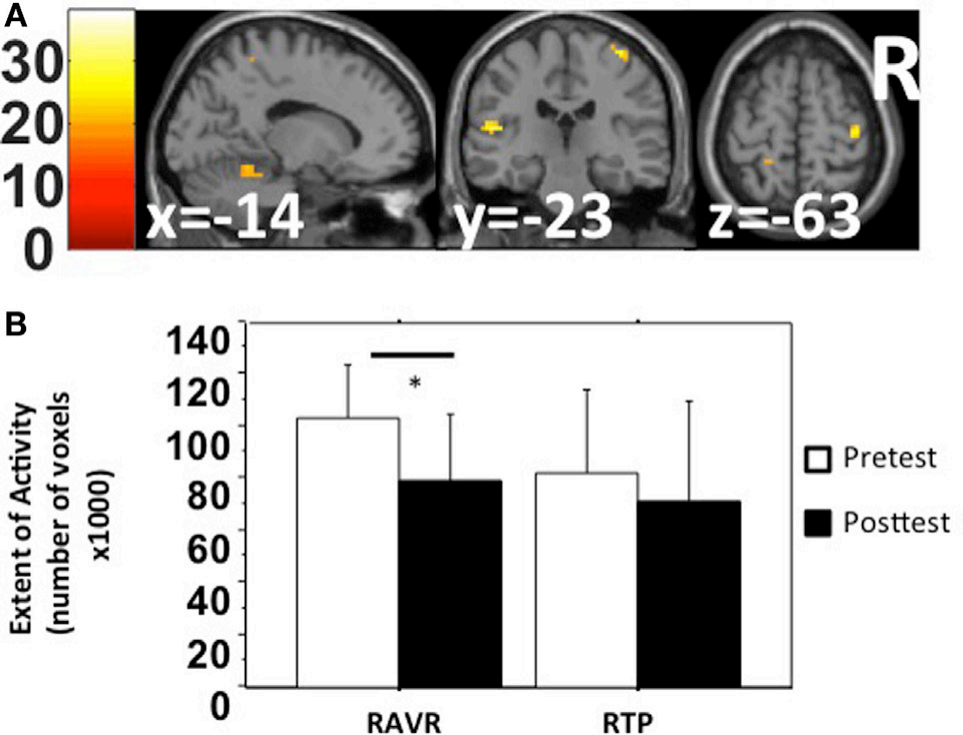
**(A)** Within-subject non-parametric ANOVA test comparing pre-test and post-test functional magnetic resonance imaging BOLD signals (robot-assisted virtual reality group, family-wise error corrected *p* < 0.05). **(B)** Extent of activity in the gray matter, in terms of number of active voxels, in both groups sorted based on group and time.

The SwE toolbox (53), for non-parametric contrast comparisons, was used to analyze changes in activation magnitude for factors: training group and test time. The activation magnitude in the cerebellum, left temporal lobe, and right precentral gyrus was significantly reduced after RAVR-based training (Figure 2A, Table 3) but did not change in the RTP group (not shown). Figure 2B shows that there was also a significant decrease in the extent of activity in the RAVR group after training (Mann–Whitney *U* test, *Z* = −2.894, *p* = 0.0038) but did not significantly change in the RTP group (Mann–Whitney *U* test, *Z* = −1.04, *p* = 0.296).

**Table 3:** Functional magnetic resonance imaging (fMRI) results of robot-assisted virtual reality group effect of interest contrast (non-parametric within-subject comparison).

Region	Side	*F* statistic	*p* value [family-wise error (FWE) corrected]	*x*, *y*, *z*	*k*
Mid. temporal l.	L	37.91	0.0059	−45 −57 −3	9
Cerebellum (Culmen)	L	36.1	0.0059	−39 −51 −30	27
Precentral g.	R	35.19	0.0059	39 −21 66	18
Sup. temporal	L	29.9	0.0137	−48 −24 12	52
Postcentral g.	L	25.9	0.0234	−48 −18 33	15
Cerebellum (Declive)	L	25.5	0.0234	−27 −54 −21	90

*Significant clusters that are FWE corrected (*p* < 0.05; extent >10) at the voxel level. Note that “right” corresponds to the ipsilesional side after flipping all patients with left-sided lesions to the right side (to allow group-level comparisons). fMRI, functional magnetic resonance imaging; *k*, cluster size in voxels; *x, y, z*, coordinates (mm) of the peak voxel in the Montreal Neurological Institute space. In column 1, l. stands for lobule, g. stands for gyrus, mid. stands for middle, and sup. stands for superior*.

#### Interhemispheric Dominance

**Figure 3:**
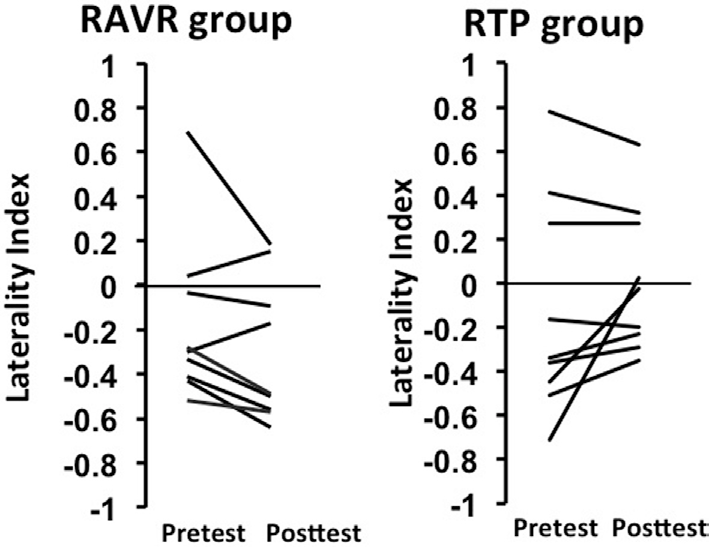
Change in laterality index in the repetitive task practice and the robot-assisted virtual reality groups.

Interhemispheric dominance (44, 45), also referred to as a LI, represents a measure of which hemisphere is predominantly active over the other hemisphere, with a value of 0 representing equal activation, positive values representing greater activity contralesionally, and negative values representing greater activity ipsilesionally. Figure 3 shows considerable variability at the individual subject level in the pre-to-post changes in interhemispheric dominance; some subjects exhibited decreases in dominance of the contralesional hemisphere, while others exhibited increases in dominance of the ipsilesional hemisphere. This result is noteworthy, however, in the sense that both patterns represent a reduction of the dominance in the contralesional hemisphere—either bringing the balance closer to 0 between the two hemispheres after training or facilitating the dominance of the ipsilesional hemisphere. The change in LI after training was significantly different between groups (RAVR group LI median = −0.125, RTP group LI median = 0.07, *Z* = −2.21, *p* = 0.027), knowing that the baseline LI values were not significantly different between groups (*Z* = −1.68, *p* = 0.09).

#### Change in Functional Connectivity with iMC

The connectivity maps with iMC as the seed region exhibited increases and decreases across different subjects. No significant difference between groups, or between the two testing days for both groups, and the pattern of change in the connectivity map for iMC was not related to lesion side (right or left), lesion site (cortical or subcortical), or impairment severity.

#### Change in Effective Connectivity

**Figure 4:**
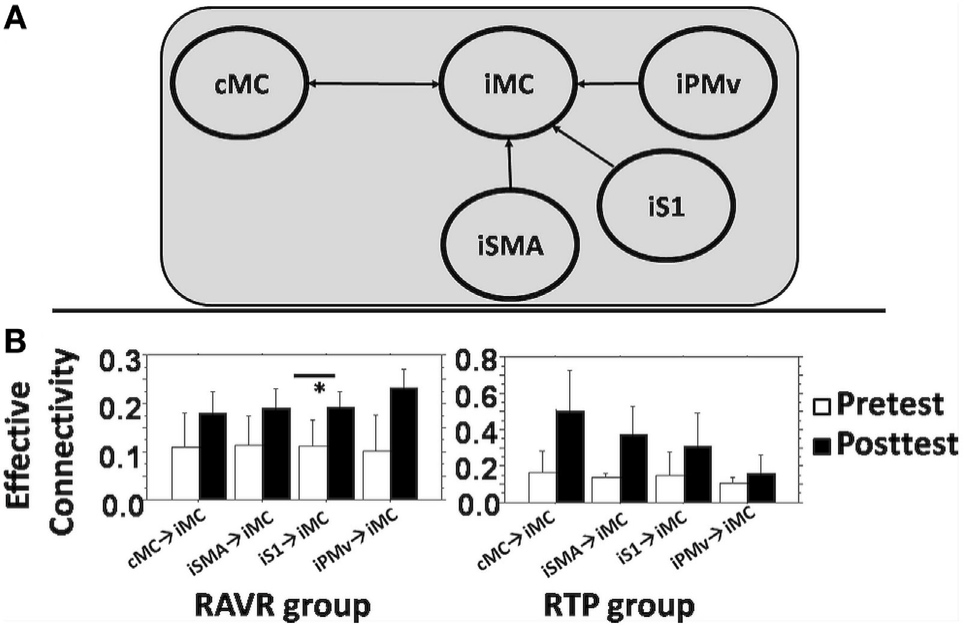
**(A)** DCM full model used to study connectivity between contralesional motor cortex, iS1, ipsilesional supplementary motor area, iPMv, and iM1. **(B)** Average change in DCM parameters (A + B) in both groups for each connectivity edge. Error bars show the SD. * denotes statistically significant difference.

DCM parameters showed varying patterns of change across subjects of both groups. Figure 4A shows the DCM model that was tested for the effective connectivity analysis. Figure 4B shows the change in DCM parameters for both groups, including connectivity parameters from cMC, iPMv, iSMA, and iS1 to iMC. In the RAVR group, DCM connectivity parameters became consistently facilitatory subsequent to training, with facilitation of iMC by iS1 significantly increasing after training (*Z* = −2.07, *p* = 0.038). In the RTP group, none of the effective connectivity parameters changed significantly after training.

### Relationship between Neurophysiological and Clinical Measures

#### Relationship to Activation Extent

**Figure 5:**
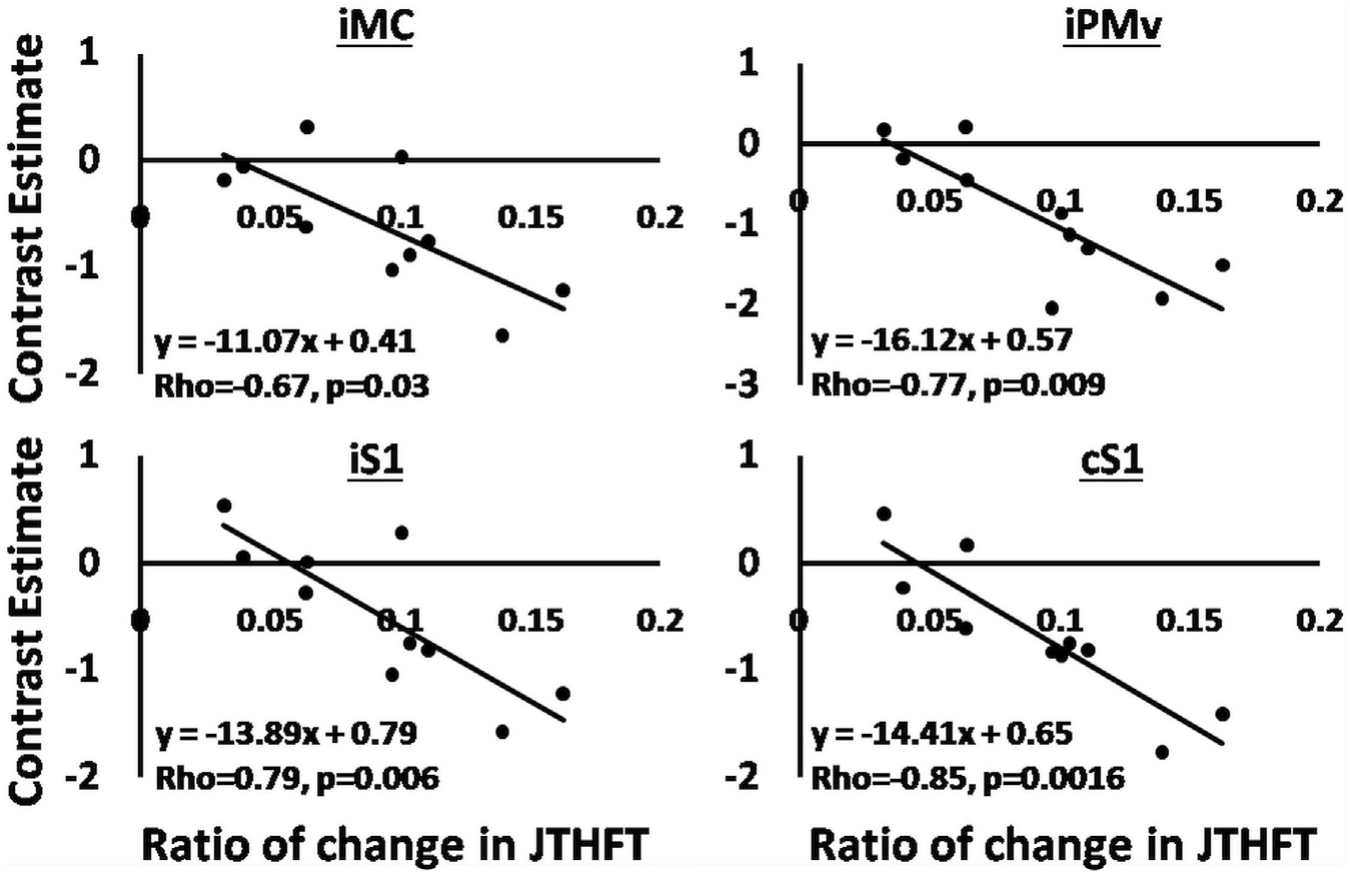
Regression analysis, the correlation between changes in BOLD signal beta signals, and ratio of change in Jebsen Taylor Hand Function Test in the robot-assisted virtual reality group. Solid lines show the fit of the data to shown equations.

We explored the relationship between clinical outcomes and magnitude of brain activation for both groups by performing a regression analysis (using non-parametric Spearman rank correlation) between the ratio of change in the beta values of brain activation for seven ROIs (bilateral premotor, sensory, and motor areas and iSMA) and the ratio of change in JTHFT scores, final JTHFT scores after training, and lesion volume. The ROIs were selected based on the main contrast (move versus rest, combining both testing sessions) and on their anatomical location and role in sensorimotor control. We noted a significant correlation in the RAVR group but not in the RTP group for iMC (rho = −0.78, *p* = 0.0061), ventral premotor area (iPMv) (rho = −0.77, *p* = 0.009), and bilateral primary sensory areas [iS1 (rho = −0.79, *p* = 0.0006) and cS1 (rho = −0.85, *p* = 0.006)] (see Figure 5).

#### Relationship to Interhemispheric Dominance

Combining the RAVR and RTP group data showed a positive correlation between the shift in LI toward the ipsilesional hemisphere (difference in LI between post-test and pre-test) and improvement in the clinical score (rho = 0.47, *p* = 0.048). The relationship between interhemispheric dominance and the change in JTHFT was not statistically significant within each group.

#### Relationship to Functional and Effective Connectivity of iMC

We found no significant relationship between the change in functional connectivity with iMC and the change in JTHFT after training, or with the lesion volume in both groups. Similarly, we found no significant correlation between the changes in DCM parameters and change in JTFHT. However, we did note that the change in DCM parameters from iS1 to iM1 significantly correlated with the post-test JTFHT score (rho = 0.73, *p* = 0.03), suggesting that there may be some importance to the connection between sensory and motor cortex in predicting the level of recovery.

## Discussion

The aim of this study was to investigate the effect of robot-assisted VR training, compared to RTP, on patterns of brain reorganization. Both training groups showed similar levels of functional recovery, consistent with other published studies comparing VR with other interventions (8, 54, 55). We hypothesized, however, that VR may engage different neural networks than RTP training and our hypothesis was partially supported. In the following sections, we discuss the results of each fMRI outcome measure (extent and magnitude of activity, lateralization, functional connectivity of iMC, and effective connectivity) and its relationship with change in clinical outcome measures.

### Extent and Magnitude of BOLD Activity

Our data revealed a decrease in magnitude and extent of brain activation in the RAVR group that correlated with improvement in the JTHFT clinical score. Similar findings have been noted by others. For example, Johansen-Berg et al. (56) showed a positive correlation between motor improvement (using grip strength as outcome measure) and *Z* stats of magnitude of activity in premotor cortex, and sensory areas [see also Ref. (57–59)]. It has been suggested that a reduction in brain activity that is observed after training might be attributed to the diminished overactivation such that less substitution is required to compensate for the lost function of injured areas (60, 61).

### Interhemispheric Dominance

We noted a difference between the groups in the change in LI after training. The change was higher in the RAVR group, shifting toward more dominance of the ipsilesional hemisphere. The above change is consistent with what is typical observed with regard to interhemispheric balance over recovery. Specifically, in the early stages following a stroke, the shift in dominance toward the contralesional hemisphere is thought to arise because of compensatory processes that allow the non-lesioned hemisphere to compensate (59, 62). Over the course of recovery, contralesional dominance tends to decrease, or shifts toward the ipsilesional hemisphere (58, 63). Interestingly, RTP training did not lead to a pronounced change in interhemispheric dominance, suggesting that the greater visuomotor feedback received by the subjects in the RAVR group may have facilitated a shift of activation back to the lesioned hemisphere.

### Functional and Effective Connectivity

Our DCM results, showing a strengthening between the iS1 and iMC ROIs, are consistent with (64), who also identified an association between higher levels of sensorimotor processing and higher levels of motor performance in persons with stroke. It is interesting that we did not note significant changes in connectivity between other nodes and iMC, for example, in contrast to Ref. (65) who noted a correlation between iSMA → iMC and cM1 → iM1 connectivity and hand performance, or Ref. (66) who noted a correlation between changes in iSMA → iMC and iPMv → iMC and motor recovery. It is possible that this difference in results between our study and those mentioned earlier may be due to the difference in the motor task used—single joint finger movement in the case of Grefkes et al. (65) and a naturalistic task that is part of the JTHFT used in our study, which may be characterized by greater variability of performance; and a difference in the stage of stroke—which <32 weeks in the case of Rehme et al. (66) and >36 weeks in the case of our study. The complex relationship between changes in the network and recovery and how this interacts with task and stage of recovery is an important issue that warrants significantly more investigation.

### Study Limitations

One of the limitations of this study is the heterogeneity of the subject pool in terms of lesion characterization, time since stroke, and motor performance. While the sample heterogeneity may have made our results more generalizable, it likely also added variability to all of the outcome measures. Another limitation is that subjects were not randomly assigned to groups. The clinical results of that investigation have been published (8). While we acknowledge that random assignment would have been preferable, the clinical measures were similar at baseline for both groups. Any differences in brain activation patterns were unlikely to be explained potential differences in baseline status since these were consistent between groups. In addition, regression analysis was performed using several parameters (lesion volume, JTHFT after training, and ratio of change in JTHFT), but the lack of correlation between some of the neurophysiological variables and these measures might be due to the small sample size, the choice of ROIs, or the measure of recovery. While functional and effective connectivity measures were used to understand dynamic changes in neural networks after training, we did not acquire DTI tractography measures and resting-state functional connectivity, and this limitation hinders the ability to relate neurophysiological changes to cortico-spinal structural connectivity and interhemispheric and intra-hemispheric resting-state functional connectivity (67). Nonetheless, though it is tempting to attribute changes in neural reorganization to the intervention, these results should be interpreted with caution until larger scale RCT designs confirm these effects.

## Conclusion

We found different patterns of reorganization for each group, with the RAVR group changes correlating with better improvement on clinical measures. The patterns of brain reorganization suggest that clinical improvement in the RTP group might have been driven by an adaptive compensatory process in the contralesional hemisphere, while improvement in the RAVR group may have been attributed more to reinstatement of activity of ipsilesional sensorimotor networks. As is often the case, there was no consistent single pattern of recovery exhibited by every subject, highlighting the need to understand patient-specific effects of interventions. However, the neural reorganization that was observed in the RAVR group was generally in line with expected patterns of recovery and suggests that VR and robotic assisted training may be a viable means of providing therapy to reinstate desirable changes in the brain.

## Ethics Statement

All subjects provided written and verbal informed consents approved by Institutional Review Boards of the New Jersey Institute of Technology and Rutgers University prior to participating.

## Author Contributions

SS did the fMRI data collection and data analysis and worked with SA and ET on disseminating the findings and with GF, SA, and ET on writing the manuscript. GF was involved in recruiting and screening participants, in administering the training interventions, collecting clinical data, and writing the manuscript. QQ performed the design and implementation of the virtual reality video games used in the interventions and in administering the training interventions. AM and SA were involved in designing and supervising the training interventions. ET designed the protocol of fMRI testing and was involved in data collection and data analysis and helped in disseminating the findings and in writing the manuscript.

## Conflict of Interest Statement

The authors declare that the research was conducted in the absence of any commercial or financial relationships that could be construed as a potential conflict of interest.
